# Prevalence and course of disease after lung resection in primary ciliary dyskinesia: a cohort & nested case-control study

**DOI:** 10.1186/s12931-019-1183-y

**Published:** 2019-09-18

**Authors:** Panayiotis Kouis, Myrofora Goutaki, Florian S. Halbeisen, Ifigeneia Gioti, Nicos Middleton, Israel Amirav, on behalf of the Israeli PCD Consortium, Angelo Barbato, on behalf of the Italian PCD Consortium, Laura Behan, Mieke Boon, Nagehan Emiralioglu, Eric G. Haarman, Bulent Karadag, Cordula Koerner-Rettberg, Romain Lazor, on behalf of the Swiss PCD Group, Michael R. Loebinger, Bernard Maitre, on behalf of the French Reference Centre for Rare Lung Diseases, Henryk Mazurek, Lucy Morgan, Kim Gjerum Nielsen, Heymut Omran, Ugur Özçelik, Mareike Price, Andrzej Pogorzelski, Deborah Snijders, on behalf of the PCD Italian Consortium, Guillaume Thouvenin, on behalf of the French Reference Centre for Rare Lung Diseases, Claudius Werner, Zorica Zivkovic, Claudia E. Kuehni, Panayiotis K. Yiallouros

**Affiliations:** 1 Respiratory Physiology Laboratory, Medical School, University of Cyprus, Nicosia, Cyprus; 2 Institute of Social and Preventive Medicine, University of Bern, Bern, Switzerland; 3 Department of Nursing, School of Health Sciences, Cyprus University of Technology, Limassol, Cyprus; 4 Department of Pediatrics University of Alberta Edmonton, Edmonton, Canada; 5 Primary Ciliary Dyskinesia Centre, Department of Women’s and Children’s Health (SDB), University of Padova, Padova, Italy; 6 Primary Ciliary Dyskinesia Centre, University Hospital Southampton, NHS Foundation Trust and University of Southampton, Southampton, UK; 7 Department of Paediatrics & Paediatric Pulmonology, University Hospital Gasthuisberg Leuven, Leuven, Belgium; 8 Pediatric Pulmonology, Hacettepe University, Ankara, Turkey; 9 Department of pediatric pulmonology, Emma Children’s Hospital, Amsterdam UMC, Vrije Universiteit, Amsterdam, The Netherlands; 10 Department of Pediatric Pulmonology, Marmara University, School of Medicine, Istanbul, Turkey; 11 Department of Paediatric Pulmonology, University Children’s Hospital of Ruhr University Bochum, Bochum, Germany; 12 Department of Respiratory Medicine, Lausanne University Hospital, Lausanne, Switzerland; 13 Department of Respiratory Medicine, National Reference Centre for Rare Pulmonary Diseases, Lyon, France; 14 Host Defence Unit, Royal Brompton and Harefield NHS Foundation Trust, London, UK; 15 Hopital intercommunal de Créteil, Service de Pneumologie, DHU ATVB, Université Paris Est Créteil, Paris, France; 16 Klinika Pneumonologii i Mukowiscydozy, Instytut Gruźlicy i ChoróbPłuc, Rabka, Poland; 17 Department of Respiratory Medicine, Concord Hospital Clinical School, University of Sydney, Sydney, Australia; 18 Danish PCD Centre Copenhagen, Paediatric Pulmonary Service, Copenhagen University Hospital, Copenhagen, Denmark; 19 Department of General Paediatrics and Adolescent Medicine, University Hospital Muenster, Muenster, Germany; 20 Clinic for Paediatric pulmonology, Allergiology and Neonatology, Hannover Medical School, Hannover, Germany; 21 Service de pneumologie pédiatrique, Hôpital Trousseau, APHP, Sorbonne Université, INSERM, Centre de Recherche Saint-Antoine, CRSA, Paris, France; 22 Children’s Hospital for Lung Diseases and TB, Medical Centre “Dr Dragisa Misovic”, Belgrade, Serbia; 23 Faculty of Pharmacy Novi Sad, Business Academy in Novi Sad, Novi Sad, Serbia; 24 Shakolas Educational Center of Clinical Medicine, Palaios Dromos Lefkosias-Lemesou 215/6,2029 Aglantzia, Nicosia, Cyprus; 25 Dana-Dwek Children’s Hospital, Tel Aviv Medical Center, Tel Aviv, Israel; 26 Department of Pediatrics, Helios Hospital Schwerin, Schwerin, Germany

**Keywords:** Kartagener syndrome (MeSH), Ciliary motility disorders (MeSH), Lobectomy

## Abstract

**Background:**

Lung resection is a controversial and understudied therapeutic modality in Primary Ciliary Dyskinesia (PCD). We assessed the prevalence of lung resection in PCD across countries and compared disease course in lobectomised and non-lobectomised patients.

**Methods:**

In the international iPCD cohort, we identified lobectomised and non-lobectomised age and sex-matched PCD patients and compared their characteristics, lung function and BMI cross-sectionally and longitudinally.

**Results:**

Among 2896 patients in the iPCD cohort, 163 from 20 centers (15 countries) underwent lung resection (5.6%). Among adult patients, prevalence of lung resection was 8.9%, demonstrating wide variation among countries. Compared to the rest of the iPCD cohort, lobectomised patients were more often females, older at diagnosis, and more often had situs solitus. In about half of the cases (45.6%) lung resection was performed before presentation to specialized PCD centers for diagnostic work-up. Compared to controls (*n* = 197), lobectomised patients had lower FVC z-scores (− 2.41 vs − 1.35, *p* = 0.0001) and FEV1 z-scores (− 2.79 vs − 1.99, *p* = 0.003) at their first post-lung resection assessment. After surgery, lung function continued to decline at a faster rate in lobectomised patients compared to controls (FVC z-score slope: − 0.037/year Vs − 0.009/year, *p* = 0.047 and FEV1 z-score slope: − 0.052/year Vs − 0.033/year, *p* = 0.235), although difference did not reach statistical significance for FEV1. Within cases, females and patients with multiple lobe resections had lower lung function.

**Conclusions:**

Prevalence of lung resection in PCD varies widely between countries, is often performed before PCD diagnosis and overall is more frequent in patients with delayed diagnosis. After lung resection, compared to controls most lobectomised patients have poorer and continuing decline of lung function despite lung resection. Further studies benefiting from prospective data collection are needed to confirm these findings.

## Background

Primary Ciliary Dyskinesia (PCD) is a genetically heterogeneous disorder characterized by laterality defects and recurrent respiratory infections [1]. Bronchiectasis may develop already in childhood [2] and it is usually present in most adult PCD patients [3]. Late diagnosis is associated with worse clinical picture, [4–6] although even early diagnosis is followed by variable courses of lung function that are not linked to the level of lung function at diagnosis [7]. Management varies considerably between centers, as there are no evidence-based therapeutic guidelines for PCD [8]. Most of the current treatment protocols are extrapolated from Cystic Fibrosis (CF) studies.

Historically, in CF patients with localized bronchiectasis, lung resection has been proposed as a measure to decrease infection burden and limit the damage to the remaining lung [9–11]. However, a recent study from the US showed no improvement in lung function, hospital admissions or antibiotic use in CF patients after lung resection [12]. Similarly, a retrospective, small-scale study in children with non-CF bronchiectasis concluded that surgical treatment did not affect annual exacerbation rates and lung function but resulted in reductions the need for annual intravenous antibiotics [13]. Lung resection to treat PCD lung disease is generally not recommended, although it can be considered for selected cases of severe and localized bronchiectasis with recurrent suppurative infection, hemoptysis or specific infection [14]. Nevertheless, this approach remains controversial and to date, only two reports described the clinical course in PCD patients after lung resection, [5, 15] with conflicting results. The older study by Smit et al. compared 13 adult lobectomised PCD patients to 8 adult non-lobectomised PCD patients and did not find significant differences in respiratory symptoms between the two groups. Despite this, 85% of the lobectomised patients subjectively perceived the operation as beneficial [15]. A more recent study from Cyprus, compared 5 lobectomised PCD patients with 7 age-matched non-lobectomised PCD patients and reported that lobectomised patients had a more severe clinical picture and consistently lower lung function across time compared to non-lobectomised patients [5]. Both reports were single-center studies with small sample size and generalizability of their results is limited.

Ideally, the performance of a carefully conducted prospective study would be required to assess the impact of lung resection in PCD patients. However, due to the low prevalence of PCD and the rarity of lung resection, this approach is unrealistic. Alternatively, analysis of retrospective international registry data could offer important answers to this question. We used the international PCD cohort (iPCD) [16] to assimilate information from a large number of PCD patients across specialized centers in order to a) assess the prevalence of lung resection among PCD patients, b) compare characteristics of lobectomised and non-lobectomised patients in the iPCD cohort and c) in a nested case-control study with more detailed data describe and identify predictors (sex, extent of lung resection, lung function prior lung resection) of disease course after lung resection.

## Methods

### Population and study design

**Fig. 1:**
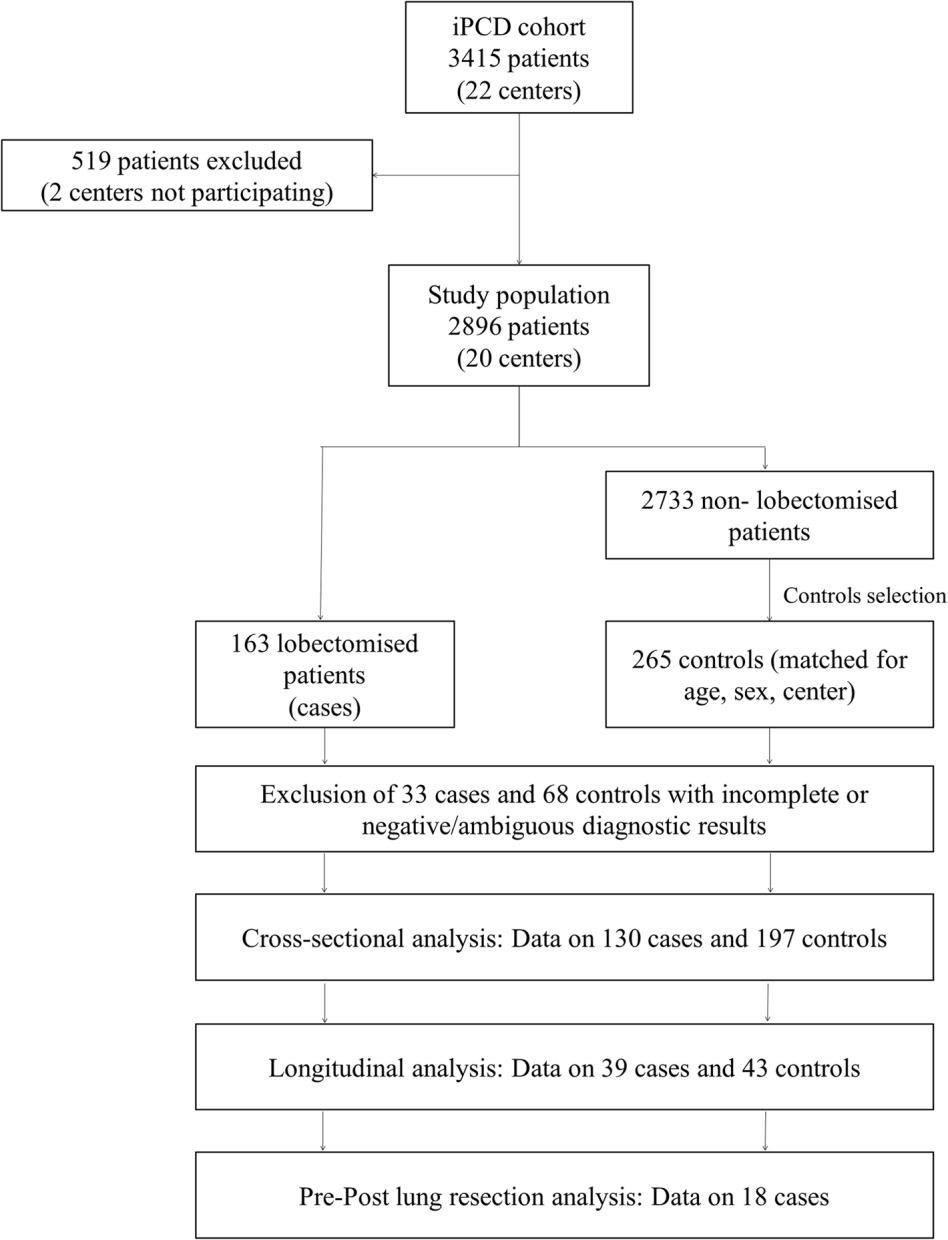
Participants selection and data availability. Flowchart of participants’ selection and data availability**.** From the 22 centers (3415 patients) that were included in the iPCD cohort, 20 centers (2896 patients) agreed to participate and provided data to the study. Of these 163 were patients that underwent lung resection. Of the remaining 2733 patients, we randomly selected 265 controls stratified by age (± 5 years), sex and center. Data availability for cross-sectional and longitudinal case-control analysis as well as data availability for the within cases pre-post lung resection analysis is also displayed

We used a dataset from the iPCD cohort, a large retrospective international cohort study of > 3000 PCD patients. All patients included in the iPCD cohort had diagnoses of other chronic lung diseases such as cystic fibrosis and primary immunodeficiency excluded. Additional details on iPCD cohort can be found elsewhere [16]. The cohort analysis included all patients whose data on lung resection status were available by May 2017. For each identified lobectomised patient, we randomly selected up to two controls, where available, stratified by age (±5 years), sex and center. In this manner, the groups of cases and controls were characterized by similar distributions over different levels of potential confounding variables such as age, sex and center (frequency matching design) [17]. Selection of cases and controls is presented in detail in Fig. 1. Information on available patient data and measurements as well as ethical approvals permitting the use of patient data is available in the online data supplement (Additional file 1).

### PCD diagnosis

The iPCD Cohort includes data from patients diagnosed as early as 1964. Since then, availability of diagnostic tests and diagnostic criteria for PCD have evolved considerably. Originally, diagnosis was largely based on the presence of the Kartagener triad (bronchiectasis, sinusitis and situs inversus) and on transmission electron microscopy (TEM) findings. With time, High Speed Video Microscopy (HSVM), as well as nasal nitric oxide (nNO) and genetic analysis were introduced in the diagnostic work-up for PCD [18]. Nevertheless, even in recent years there is considerable variability in the availability of these tests between countries [8]. Towards better defining our study population, we classified all identified lobectomised patients and PCD controls in three diagnostic groups according to the recent guidelines of the ERS PCD Diagnostics Task Force [19]. More specifically, we classified PCD patients a) as “definite PCD” if they had hallmark EM findings and/or biallelic PCD genetic mutations, b) as “PCD highly likely” if they had abnormal HSVM findings and/or low nNO (using a cut-off of 77 nl/min as suggested by Leigh MW et al., 2013), and c) as “clinical PCD” if they had a clinical phenotype suggestive of PCD but the PCD diagnostic algorithm was incomplete or diagnostic results were negative or ambiguous. All patients classified as “clinical PCD” were excluded from statistical analysis and both the cross-sectional and longitudinal analysis is based on patients classified only as “definite PCD” or “PCD highly likely”.

### Analysis

#### iPCD cohort study

The prevalence of lung resection in PCD was calculated as percentage of all PCD patients in the dataset, as well as percentage of all PCD patients per study center. Similarly, prevalence of lung resection among adults (≥18 years) with PCD was calculated as percentage of total adult PCD patients in the dataset and per country. Basic characteristics of lobectomised patients were summarized as percentages or as medians and inter-quartile ranges (IQR), as appropriate. Differences in the distribution of characteristics between lobectomised and non-lobectomised patients in the iPCD cohort were investigated using chi-square test for categorical variables and Wilcoxon Sum Rank test for continuous variables.

#### Nested case-control study - cross-sectional comparisons

Diagnostic results and clinical characteristics of lobectomised patients (at first available post-lobectomy assessment) and non-lobectomised controls were compared using paired sample t-test and Wilcoxon Sum Rank test for normally and non-normally distributed variables respectively.

#### Nested case-control study – longitudinal comparisons

We explored disease course post-lung resection in lobectomised PCD patients compared to controls, using serial measurements of FEV_1_, FVC and BMI in a longitudinal mixed effects model. The model was defined by fixed effects for lung resection and age, an interaction term between lung resection and age and by random effects for intercepts and slopes (change per year). In addition, the frequency of positive sputum cultures for *Pseudomonas aeruginosa* was compared between cases and controls across different age groups (5–10, 10–15, 15–20, 20–30 and 30+) by chi square test.

#### Predictors of disease course within cases – longitudinal comparisons

We also explored the effect of sex and number of lobes resected as potential predictors of adverse clinical course within the lobectomised group with two separate subgroup analyses. In the first subgroup analysis by sex, serial measurements of FEV1, FVC and BMI were analyzed with longitudinal mixed effect model defined as above and using a binary fixed effect term for sex while in the second subgroup analysis, the longitudinal mixed effect model used a binary fixed effect term for number of lobes resected.

#### Pre- and post- lung resection comparisons

In a small subgroup of lobectomised patients, we analyzed available lung function and BMI data before and after lung resection with the aim to provide insight to the critical question whether the adverse disease course of lobectomised patients is due to lung resection or to more severe disease phenotype prior lung resection. FEV_1_, FVC and BMI z-scores were plotted across time and the mean pre- and post- lung resection values were compared using paired samples t-test. Furthermore, mean lung function z-scores of lobectomised patients at their last assessment prior to lung resection were compared with the mean of lung function measurements in controls obtained when they had the same age, using two-way analysis of variance to adjust for the effect of age. Lastly, the longitudinal mixed model analysis was repeated for this small group of patients and for an equal number of controls matched based on propensity score calculated using the variables for level of diagnostic certainty, sex, age at presentation and positive sputum culture. The calculation of the propensity score was carried out using the STATA “pscore” command [20].

Pairwise deletion was used to handle missing data in an analysis by analysis basis. Statistical comparisons were performed using STATA 12 (StataCorp, TX) and graphs were generated with Microsoft Excel.

## Results

### iPCD cohort study - prevalence of lung resection and patient characteristics

**Fig. 2:**
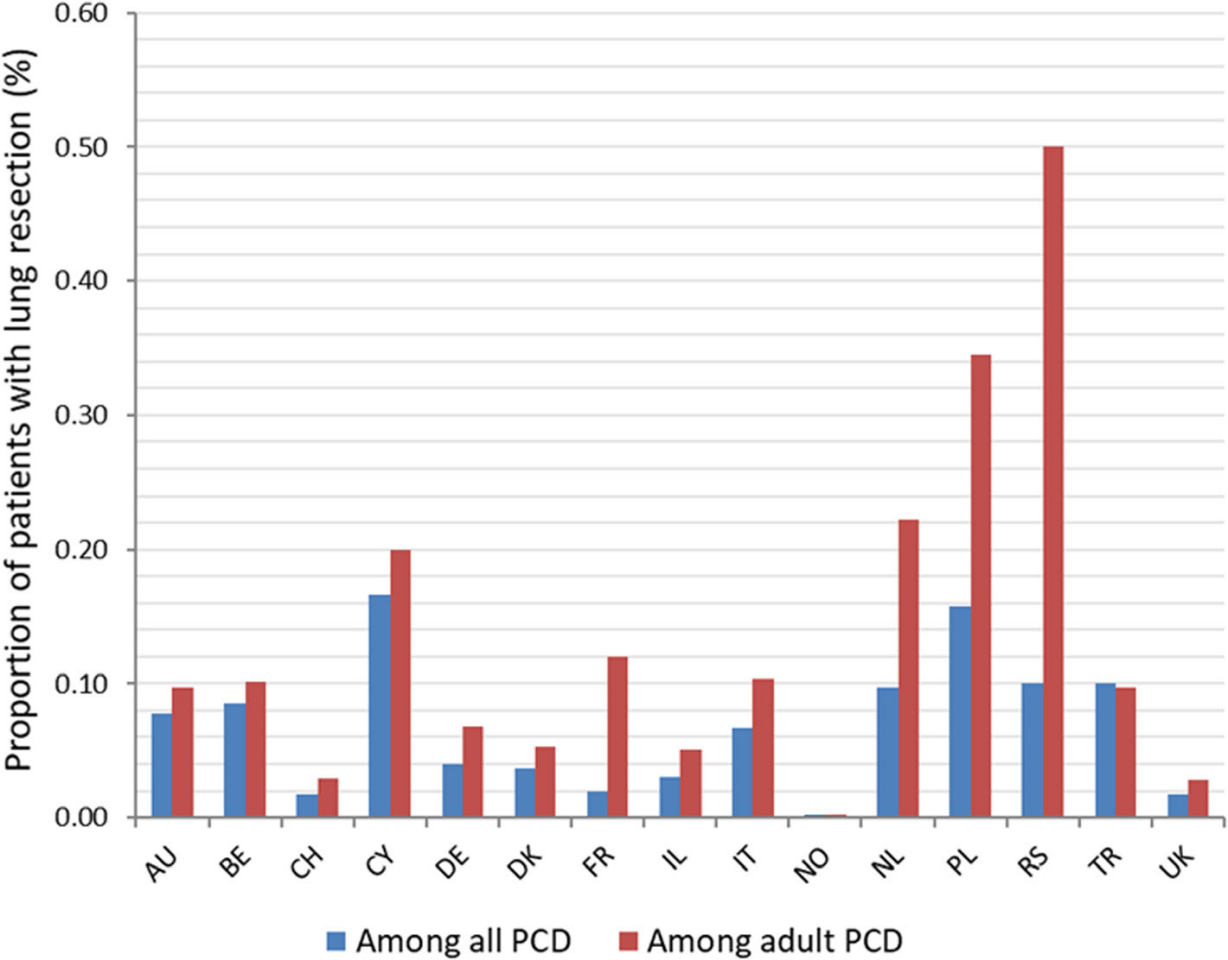
The frequency of lung resection among patients in the iPCD cohort by participating centers. The prevalence of lung resection among PCD patients in the iPCD cohort across different centers. Prevalence among all PCD patients is denoted with dark color and prevalence among adult PCD patients is denoted with lighter pattern color. Absolute numbers are displayed in Additional file 1: Table S1 (Additional file 1). AU: Australia; BE: Belgium; CH: Switzerland; CY: Cyprus; DE1: Bochum, Germany, DE2: Muenster, Germany; DE3: Hannover, Germany; DK: Denmark; FR: France; IL: Israel; IT: Italy; NL: the Netherlands; NO: Norway; PL: Poland; RS: Serbia; TR1: Istanbul, Turkey; TR2: Ankara, Turkey; UK1: Paediatric Pulmonology Dept, Brompton, UK; UK2: Adult Pulmonology Dept, Brompton, UK; UK3: Southampton, UK

From 2896 PCD patients, followed-up in 20 centers across 15 countries, 163 (5.6%) were reported to have undergone lung resection (Fig. 1). Among adults with PCD, lung resection was reported for 127 patients out of 1431 (8.9%), whereas in the pediatric age-group only 36 out of 1465 (2.5%) underwent surgical treatment. Wide variation was observed in lung resection prevalence among PCD centers (range: 0 to 17%, Fig. 2, absolute numbers in Additional file 1: Table S1).

Lobectomised patients were significantly older at the time of the study (median age 24.9 vs 18.6 years, *p*-value< 0.001) and included more females (55.4% vs 49.0%, *p*-value = 0.150) than the non-lobectomised iPCD cohort (Table 1). In addition, lobectomised patients presented to the PCD specialist centers at an older age (11.5 vs 8.8 years, *p*-value< 0.001) and had less frequently laterality defects (25% vs 44.1%, *p*-value< 0.001) than the rest of the cohort. The number of lung resection procedures performed per decade in iPCD cohort patients seemed to be increasing (*p*-value < 0.001) in the last decades (Table 1). In most cases (83.5%), lung resection was performed when the patients were in childhood and in about half of the cases (45.6%) lung resection was performed before presentation to the specialized PCD centers for diagnostic work-up. Stratification by lung resections performed before or after presentation to a PCD center demonstrated that before 1990, almost all lung resections occurred prior presentation to a PCD center (100% < 1969, 73% during 1970–1979 and 89% during 1980–1989). During the period 1990 to 2010, approximately 50% of lung resections occurred prior presentation to a PCD center, as opposed to only 18% after 2010 (Additional file 1: Table S3). In a quarter of the cases (22.1%), lung resection was extended beyond one lobe, involving segments in 2 to 4 lobes, whereas the most frequently resected segments were at the right middle lobe (48.8%) and left lower lobe (27.3%) (Table 1).

**Table 1:** Features of lobectomised and non-lobectomised patients in the iPCD cohort

Variable	Lobectomised PCD (*n* = 130) Median (IQR)		iPCD Cohort (*n* = 2733)^a^ Median (IQR)	*p*-value
Age at Presentation (*n* = 118)	11.5 (6.5, 20.5)		8.8 (4.0, 15.8)	< 0.001†
Current Age (*n* = 130)	24.9 (18.1, 40.1)		18.6 (12.1, 28.3)	< 0.001†
% Female	72/130 (55.4%)		1302/2661 (48.9%)	0.150‡
Situs Inversus	27/108 (25.0%)		787/1783 (44.1%)	< 0.001‡
Age at lung resection (*n* = 121)	11.9 (7.7, 16.0)			
Lung resection prior to presentation	52/114 (45.6%)			
Lung resection in childhood	101/121 (83.5%)			
Frequency of lung resection performance per decade (*n* = 121)	< 1969	4		< 0.001‡
	1970–1979	11		
	1980–1989	9		
	1990–1999	24		
	2000–2009	34		
	2010–2017	39		
Extent of lung resection (*n* = 95)	One Lobe: 74/95			
	(77.9%)			
	Two lobes: 17/95			
	(17.9%)			
	Three Lobes: 2/95			
	(2.1%)			
	Four Lobes: 2/95			
	(2.1%)			
Site of lung resection^b^ (*n* = 95)	RML: 59/121 (48.8%)			
	LLL: 33/121 (27.3%)			
	RLL: 13/121 (10.7%)			
	Lingula: 9/121 (7.4%)			
	RUL: 5/121 (4.1%)			
	LUL: 2/121 (1.7%)			

Denominators indicate number of subjects with available data on the specific parameter

*RUL*: Right Upper Lobe, *RML*: Right Middle Lobe, *RLL*: Right Lower Lobe, *LUL*: Left Upper Lobe, *LLL*: Left Lower Lobe

^a^Full iPCD cohort that participated in the study, excluding lobectomised patients (May 2017)

^b^Denominator reflects the total number of resected lobes. Some patients had more than one lobe resected. For one patient (one with one lobe resected) the exact site was not reported

†Wilcoxon Sum Rank Test

‡Pearson Chi Square Test

### Nested case-control study – cross-sectional comparisons

In total, 265 center, age and sex-matched non-lobectomised PCD patients were selected as controls from the 20 participating centers (Fig. 1). The targeted 1:2 cases-controls ratio was not achieved for every case due to lack of eligible controls in some centers. After exclusion of patients characterized by ambiguous or incomplete diagnostic results (“clinical PCD”), a total of 130 lobectomised and 197 controls were compared. Although nasal nitric oxide was low in both cases and controls, it was somewhat higher in lobectomised patients (median: 16 vs 10 nl/min, *p*-value = 0.013). Ciliary ultrastructure and motility were not different between the two groups (Additional file 1: Table S2). Cross-sectional comparisons of clinical characteristics of controls at presentation with those of lobectomised patients at their first available post-lobectomy assessment, revealed higher prevalence of bronchiectasis (95.7% vs 76.1%, *p*-value = 0.004) and chronic cough (96.8% vs 88.4%, *p*-value = 0.023) but less wheezing (39.7% vs 59.2%, *p*-value = 0.016) in the lobectomised cohort (Table 2). Compared to controls, lobectomised patients had lower FVC (− 2.41 vs − 1.35, *p* = 0.0001) and FEV_1_ (− 2.79 vs − 1.99, *p* = 0.003) z-scores at baseline (first post-lobectomy assessment) but no difference in BMI z-scores (0.03 vs − 0.09, *p*-value = 0.599).

**Table 2:** Cross-sectional characteristics of lobectomised PCD patients (on first post-lung resection assessment) compared to matched controls

	Lobectomised PCD (*n* = 130)	Controls PCD (*n* = 197)	*p*-value†
FVC Z score	− 2.41 (− 2.91, − 1.90)	− 1.35 (− 1.70, − 1.00)	0.0001‡
FEV1 Z score	−2.79 (− 3.25, − 2.32)	−1.99 (− 2.32, − 1.65)	0.003‡
BMI Z score	0.03 (− 0.34, 0.40)	− 0.09 (− 0.38, 0.19)	0.599‡
Sputum Culture			
Any Pathogen	60/73 (82.2%)	74/92 (80.4%)	0.774
Pseudomonas	20/73 (27.4%)	15/92 (16.3%)	0.083
Bronchiectasis	44/46 (95.7%)	86/113 (76.1%)	0.004
Congenital Heart Disease	10/86 (11.6%)	13/126 (10.3%)	0.763
NRDS	37/78 (47.4%)	51/114 (44.7%)	0.712
Chronic Cough	92/95 (96.8%)	99/112 (88.4%)	0.023
Sputum	78/82 (95.1%)	73/81 (90.1%)	0.222
Wheezing	25/63 (39.7%)	58/98 (59.2%)	0.016
Pneumonia	11/66 (16.7%)	35/120 (29.2%)	0.059
Hemoptysis	2/58 (3.5%)	0/67 (0%)	0.125
Rhinorrhea	68/79 (86.1%)	111/123 (90.2%)	0.363

Denominators indicate number of subjects with available data on the specific parameter NRDS: Neonatal Respiratory Distress Syndrome †Pearson Chi Square Test, with the exception of FVC, FEV1 and BMI ‡Paired Samples T test

### Nested case-control study – longitudinal comparisons

In a longitudinal mixed model analysis, we included 39 lobectomised PCD patients and 43 non-lobectomised controls who had available two or more repeated measurements of FVC, FEV_1_ and BMI. The mean time interval with available longitudinal measurements for each lobectomised patient was 9.20 years (95% CI: 7.40–11.01) while the mean interval for each control patient was 10.82 years (95% CI: 8.22–13.41). The difference between the two values was not statistically significant (*p*-value: 0.310). We found no difference at the level (intercept) of FVC (− 1.00 vs − 0.75, *p*-value = 0.489) and FEV_1_ (− 1.47 vs − 1.12, *p*-value = 0.346) between lobectomised and control patients (Table 3). However, both control and lobectomised groups, displayed loss of lung function with time. Although lung function decline in the lobectomised patients after lung resection was somewhat steeper in terms of FVC (z-score slope: − 0.037/year vs – 0.009/year, *p* = 0.047) and FEV_1_ (z-score slope: − 0.052/year vs − 0.033/year, *p* = 0.235), the difference in the latter did not reach statistical significance. In contrast, BMI remained stable post-lung resection in lobectomised patients compared to controls (z-score slope: 0.024/year vs 0.015/year, *p* = 0.507) (Table 3). Lobectomised patients had also more frequent (77/263, 29.3%) positive sputum cultures for *Pseudomonas aeruginosa* compared to controls (66/487, 13.6%) (*p*-value< 0.001) that was evident in age-groups after the age of 10 years (Additional file 2: Figure S1).

**Table 3:** Change in lung function and BMI over time (post-lung resection) in lobectomised PCD patients (*n* = 39) and controls (*n* = 43)

Outcome	Group	Intercept (95% CI)	*p*-value	Change per year (95% CI)	*p*-value*
FVC Z score	Lobectomised	−1.00	0.489	−0.037	0.047
		(−2.17, −0.17)		(−0.09, 0.01)	
	Controls	−0.75		−0.009	
		(−1.20, − 0.29)		(− 0.03, 0.01)	
FEV1 Z score	Lobectomised	−1.47	0.346	− 0.052	0.235
		(−2.65, −0.28)		(− 0.10, 0.00)	
	Controls	−1.12		−0.033	
				(−0.05, − 0.02)	
		(−1.58, −0.66)			
BMI Z score	Lobectomised	−0.44	0.790	0.024	0.507
		(−1.49, 0.61)		(−0.02, 0.07)	
	Controls	−0.35		0.015	
		(−0.74, 0.04)		(−0.002, 0.03)	

* *P* value for interaction, testing whether the relationship between independent variables (FVC, FEV_1_, BMI) and time is different between Lobectomised and Controls

### Predictors of disease course

Within lobectomised patients, subgroup analysis by sex indicated sharp differences between males and females in the level (intercept) of FEV_1_ (− 0.94 vs − 1.96, *p*-value = 0.064) and FVC (− 0.68 vs − 1.65, *p*-value = 0.125) z-scores after lung resection, with females having significantly worse lung function. Patients of both sexes continued to lose lung function with time after lung resection but changes were somewhat steeper in males, with significantly worse slope for FEV_1_ (− 0.08/year vs − 0.03/year, *p*-value = 0.026) but not for FVC (− 0.05/year vs − 0.021/year, *p*-value = 0.263) (Table 4). In subgroup analysis by the number of lobes resected, we found unfavorable differences at the level of FVC (− 0.75 vs − 2.49, *p*-value = 0.040) and FEV_1_ (− 1.10 vs − 2.89, *p*-value = 0.012) z-scores intercepts in patients who underwent multiple lobes resection in comparison to patients who had only one lobe resected. However, subsequent lung function decline with ageing was not different between the two groups (Table 4).

**Table 4:** Subgroup Analysis: Change in lung function and BMI over time (post-lung resection) by sex (*n* = 39) and by extent of lung resection (*n* = 35)

Outcome	Subgroup	Intercept (95% CI)	*p*-value	Change per year (95% CI)	*p*-value*
FVC Z score	Males	−0.68	0.125	−0.049	0.263
		(−1.57, 0.22)		(−0.09, − 0.01)	
	Females	−1.65		−0.021	
		(−3.79, 0.49)		(−0.11, 0.07)	
FEV_1_ Z score	Males	−0.94	0.064	−0.08	0.026
		(−1.71, −0.17)		(−0.12, − 0.05)	
	Females	−1.96		−0.03	
		(−3.81, −0.11)		(−0.11, 0.04)	
BMI Z score	Males	−0.04	0.180	0.02	0.967
		(−0.71, 0.62)		(−0.01, 0.05)	
	Females	−0.68		0.02	
		(−2.27, −0.92)		(−0.05, 0.09)	
FVC Z score	Single Lobe	−0.75	0.040	−0.03	0.645
		(−1.38, −0.13)		(−0.06, − 0.01)	
	Multiple Lobes	−2.49		−0.05	
		(−4.79, −0.21)		(−0.14, 0.04)	
FEV1 Z score	Single Lobe	−1.10	0.012	−0.06	0.955
		(−1.61, −0.60)		(−0.08, − 0.03)	
	Multiple Lobes	−2.89		−0.06	
		(−4.78, −0.99)		(−0.14, 0.02)	
BMI Z score	Single Lobe	−0.15	0.456	0.02	0.042
		(−0.64, 0.35)		(0.01, 0.04)	
	Multiple Lobes	−0.62		−0.02	
		(−2.35, 1.12)		(−0.08, 0.04)	

*P value for interaction, testing whether the relationship between independent variables (FVC, FEV_1_, BMI) and time is different within the lobectomised patients between males and females (Subgroup Analysis 1) and between single and multiple lobes resected (Subgroup Analysis 2)

**Fig. 3:**
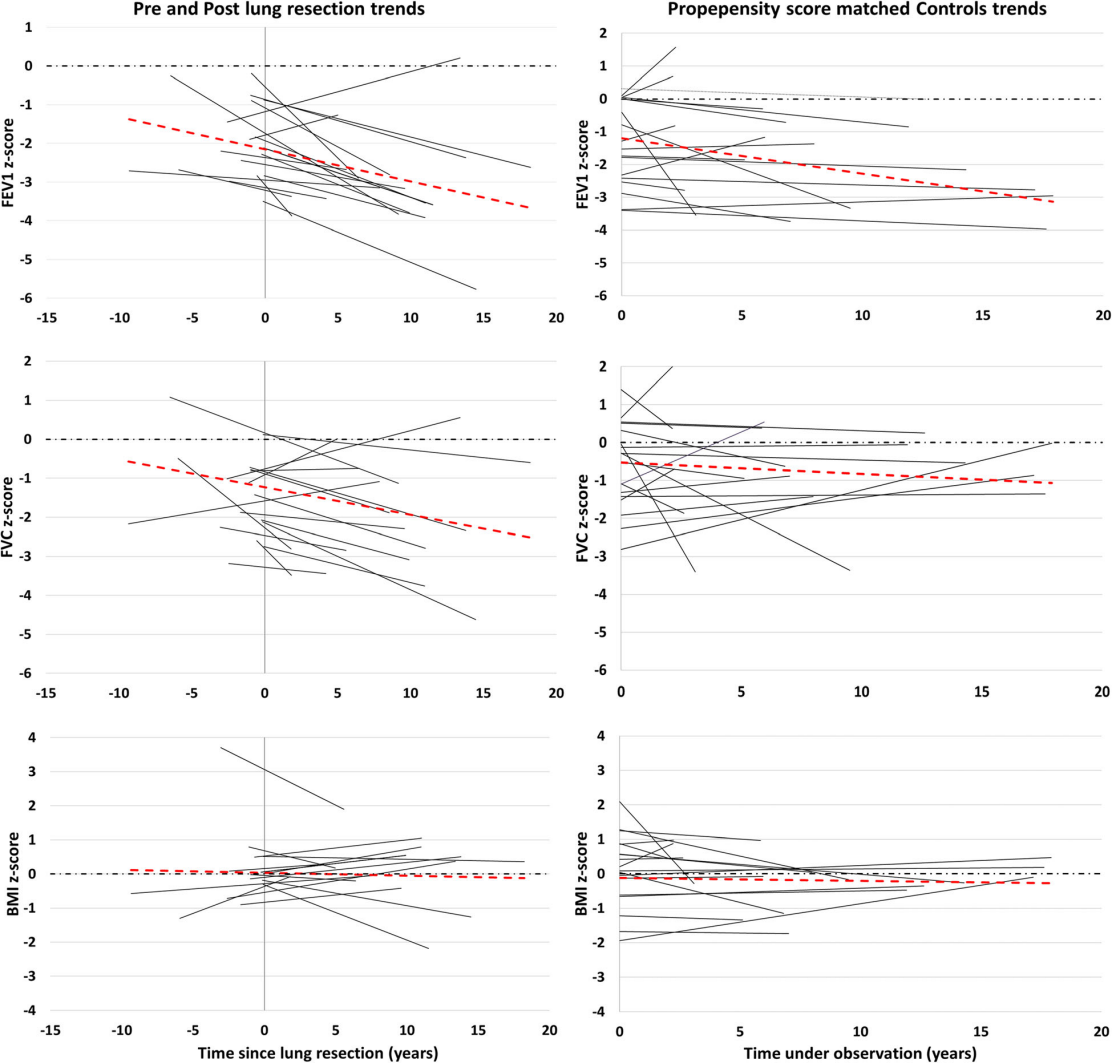
Lung function and BMI z-scores across time in PCD lobectomised patients (*n* = 18), before and after lung resection and propensity score matched controls (*n* = 18). The left panel displays the trend of lung function and BMI z-scores across time in 18 lobectomised PCD patients with available data before and after lung resection. The dashed red line represents the average trend for all 18 patients. The dashed black line denotes the zero z-score level. The overall mean pre-resection lung function z-scores were significantly higher compared to post-resection lung function z-scores (FEV1: − 1.77 Vs − 2.69 *p*-value < 0.001, FVC: − 1.16 Vs − 1.99 *p*-value: 0.003). BMI z-scores did not differ significantly (0.33Vs 0.22 *p*-value: 0.669). The right panel displays the trend of lung function and BMI z-scores across time in 18 propensity score matched controls

In a subgroup of 18 lobectomised patients, lung function and BMI data before and after lung resection were available. The basic characteristics of these 18 patients are presented in Additional file 1: Table S4. The individual FVC, FEV_1_ and BMI z-scores trends as well as the average trend for these patients across time, before and after lung resection appear in Fig. 3 in comparison to the respective trends of propensity score matched controls. Average lung function z-scores before lung resection were significantly higher when compared to z-scores after lung resection (FEV_1_: − 1.77 Vs − 2.69 *p*-value < 0.001, FVC: − 1.16 Vs − 1.99, *p*-value = 0.003), whereas BMI z-scores pre and post-lung resection did not differ (0.33 Vs 0.22, *p*-value = 0.669). Mean lung function z-scores of lobectomised patients at their last assessment prior lung resection did not differ from the mean lung function of matched non-lobectomised patients obtained when they had the same age (mean difference in FEV_1_: 0.00075, *p*-value = 0.820, mean difference in FVC: 0.34, *p*-value = 0.512). The analysis of FVC, FEV1 and BMI z-score decline between the 18 lobectomised patients and their respective propensity score matched controls demonstrated a steeper decline per year in the lobectomised patients especially in FVC z-scores (− 0.080 Vs 0.010, *p*-value = 0.006) and not so in FEV1 z-scores (− 0.099 Vs − 0.029, *p*-value = 0.071) (Additional file 1: Table S5).

## Discussion

In this study, we present the first cumulative retrospective data on lung resection in a large number (*n* = 163) of PCD patients, reported from 20 centers across 15 countries. Despite the anecdote that lobectomy is uncommon in PCD, in this representative sample of 2896 PCD patients, we report an overall prevalence of lobectomy nearly 6%, whereas in adult PCD populations from one fifth of the centers prevalence exceeds 20%. Prevalence rates of lung resection in single-center reports, [9, 10, 12] in CF cohorts are much lower (3%) compared to our series. There are no large scale, international data either on the prevalence or on long-term outcomes of lung resection in CF.

The characteristics of lobectomised patients provide possible explanations for the high prevalence of lung resection in PCD. Lobectomised PCD patients were diagnosed at an older age and had much less frequently laterality defects, indicating the difficulty to establish the diagnosis in these patients, in comparison to the rest of the iPCD cohort. In fact, in about half of the cases, lung resection was performed prior diagnosis, which suggests that in these patients the decision of lung resection was taken at a time when the nature of chronic lung disease was probably unknown. Interestingly, lung resections prior to diagnosis were performed more frequently during the earlier decades with available data (before 1990), in a period where awareness and knowledge about PCD was scarce [4]. This seems to have changed in later decades (after 1990), where the opposite trend was observed with gradually less lung resections performed prior to presentation. After 2010, it appears that very few PCD patients underwent lung resection prior to presentation (18%), although the latter figure may be an underestimation of the reality as some undiagnosed PCD patients undergoing lung resection in the last few years might have not been diagnosed by PCD centers yet. Differences in age of diagnosis as well as in surgical and medical care of bronchiectasis across countries may explain the differences in the rates of lung resection across countries. Persistent atelectasis and consolidation, especially at the middle and lower lobes, are common features in PCD [21, 22]. In comparison to CF, MRI and CT scores for lung collapse/consolidation in PCD are of higher severity, [23–25] which may be a contributing factor to the more frequent performance of lung resection in PCD. RML was by far the most frequently resected lobe, indicating the frequency and severity of the involvement of this lobe in PCD lung disease. Previous reports found associations between bronchiectasis development in RML and unfavorable clinical outcomes [26, 27].

Our study also provides important insight in PCD disease course after lung resection. Lobectomised patients have higher prevalence of bronchiectasis and lower FVC and FEV1 at first post-lung resection assessment when compared to their matched controls, as may be expected after loss of lung tissue. Despite performing lung resection to treat chronic lung disease complications, most lobectomised patients continue to display loss of lung function with time after surgery, which is somewhat steeper in terms of FVC but not FEV1, in comparison to non-lobectomised controls. Knowledge of this may have important implications at the stage of considering the decision to undertake such an intervention. This trend was also evident when we compared the course of lung function between the 18 patients that had available lung function data pre and post lung resection and 18 propensity score matched controls. Lobectomised patients also display more frequent growth of *Pseudomonas aeruginosa* in their sputa, which was shown in one adult PCD study [28] to be a poor predictor of lung function, although this was not confirmed in other studies [29]. Although a previous iPCD study [30] showed association of lung function with BMI, we found that BMI had a stable course in our lobectomised and control groups.

Patients who underwent multiple lobes resection had much lower level of lung function in comparison to those with only one lobe resected. This is not surprising given that more lung tissue was removed, but interestingly these patients display similar loss of lung function after lung resection with patients who underwent single lobe resection. Being female has been recently acknowledged as a predictor for poor lung function in adult PCD patients, [28] and it is a well-known predictor of adverse disease course in CF. [31, 32] In this series, being female is associated with adverse post-lung resection lung function, although loss of lung function in subsequent years is somewhat more profound in males who seem to lose more rapidly thereafter the excess lung function they have in comparison to females.

In a small subgroup of 18 patients, we had some indications that the mean lung function prior lung resection declined after surgery. However, lung volume reduction by the operation is confounding the value of lung function as a reliable parameter for the comparison of disease course pre and post operation. Pre-resection lung function was similar to the lung function of matched non-lobectomised PCD patients, suggesting that at least in terms of lung function the disease course before surgery was similar to non-lobectomised patients. Unfortunately, we have no detailed data on other important clinical indices prior lung resection, such as extent of bronchiectasis and clinical morbidity that are also key parameters of clinical severity. Interestingly, in contrast to the adverse lung function trend in the whole group, evaluation of individual FVC and FEV_1_ trends before and after lung resection in a minority of patients demonstrated improved lung function after surgery. This finding suggests that surgical intervention may be a worthwhile option in carefully selected PCD patients with severe localized symptomatic bronchiectasis. More studies are needed to elicit predisposing factors for the favorable post-lung resection course in this minority of patients.

The major strength of this study is the provision of data on a large, representative, international sample of PCD patients who underwent surgery to treat bronchiectasis, demonstrating continuing lung function decline after surgery. However, the study was retrospective and although effort was made to collect all relevant data based on a predefined protocol, not only from the iPCD registry but also by contacting the local principal investigators at the collaborating centers, we cannot exclude that some data were missed. In addition, given its retrospective nature, the study did not benefit from a standardized diagnostic protocol (not all PCD patients underwent the same tests across different centers) or standardized lung function testing procedures as described by the ERS/ATS recommendations [33]. The multicenter nature of the study also might introduce center-related heterogeneity in the collected parameters that we tried to control by recruiting for each lobectomised patient two controls matched for age, sex and center, although this was not possible for all cases. Furthermore, participating patients had different stages of disease and underwent different kinds of surgical intervention, introducing variabilities that may affect the outcomes, which are impossible to assess and control in a retrospective study. Overall, although this is the first study that examines and provides insights into the pragmatic outcomes of lung resection in a large number of PCD patients across many international centers, the retrospective nature of the data as well as the small sample size available for some of the subgroup analyses warrant caution in the generalization of the results.

## Conclusions

Prevalence of lung resection for treating bronchiectasis in PCD varies widely between countries and is unexpectedly high in some cohorts of adult patients. Historically, lung segments were removed from patients who were subsequently diagnosed with PCD. These patients tended to not have other clues for the diagnosis such as situs inversus. PCD diagnosis should be considered in all patients with bronchiectasis, especially those with bronchiectasis severe enough for lobectomy to be considered. This study demonstrates that lung resection in PCD, especially in female patients, is associated with more severe impairment of lung function, which continues to decline after lung resection. Further studies, benefiting from prospective data collection are needed to confirm these findings.

## Supplementary information

**Additional file 1.** Online Data Supplement (Text and Tables).

**Additional file 2: Figure S1.** Frequency of Pseudomonas positive cultures in PCD lobectomised patients (*n* = 24) and controls (*n* = 43). Frequency of Pseudomonas positive cultures among lobectomised patients (displayed in black) and among controls (displayed in grey). The proportion of positive sputum cultures for *Pseudomonas aeruginosa* in each group was calculated as the sum of individual patients’ proportions of positive cultures weighted by the ratio of the number of cultures taken from the individual patient versus the total number of cultures in the specific age group.

## Data Availability

The datasets used and analyzed during the current study are available from the corresponding author on reasonable request.
