# Supplementary material for: Prevalence and course of disease after lung resection in primary ciliary dyskinesia: a cohort & nested case-control study
Source: Respir Res. 2019 Sep 18;20:212. doi: 10.1186/s12931-019-1183-y (PMC6751891; doi:10.1186/s12931-019-1183-y)
Supplement: Supplementary file 1 — Additional file 1. Online Data Supplement (Text and Tables). [file 12931_2019_1183_MOESM1_ESM.docx]

**Online Data Supplement (Text and Tables)**

**Methods**

**Information on measurements**

Demographic data, diagnostic results, clinical characteristics, chest imaging data, lung function test results, body mass index (BMI), sputum microbiology and information regarding lung resection (date, extent, site of surgery) were extracted from patient charts and uploaded on iPCD database. Age at presentation was defined as the age at which a patient first presented at a PCD center or in case this information was unavailable, as the age of PCD diagnosis. Forced Expiratory Volume in one second (FEV1), Forced Vital Capacity (FVC) and BMI were used as primary indices of disease severity. FEV1 and FVC were expressed as age, height, ethnicity and sex adjusted z-scores based on the Global Lung Function Initiative (GLI) reference values [1]. Similarly, age- and sex-adjusted BMI z-scores were calculated based on World Health Organization (WHO) reference values [2]. Serial sputum microbiology results were also extracted from the iPCD cohort. For each patient, one sputum culture result in every three-month period (maximum 4 microbiology results per year) was included in the dataset. All local principal investigators at collaborating centers were contacted at the start of data collection and were asked to provide all relevant data based on the predefined study protocol, if these were not already available in the iPCD database.

**Information on ethical approvals**

In most participating countries, research laws do not require obtaining patients' informed consent for retrospectively collected observational data that are anonymised. However, even in these countries, local Primary Investigators (PIs) received ethics approval and informed consent for collecting patient data for local research use or national registries. In countries where informed consent is required even for anonymised observational data, PIs are responsible for obtaining ethics approval and informed consent in their country for the contribution of their anonymised data to the iPCD Cohort for research purposes. The iPCD cohort database is maintained at University of Bern (Approval Number: 060/15, Kantonale Ethikkommission für die Forschung Bern).

**References:**

1. Quanjer PH, Stanojevic S, Cole TJ, et al. Multi-ethnic reference values for spirometry for the 3-95-yr age range: the global lung function 2012 equations. Eur Respir J. 2012;40: 1324-1343.
2. Onis M. WHO Child Growth Standards based on length/height, weight and age. Acta paediatrica. 2006;95: 76-85.

**Table S1: PCD patients and lobectomised PCD patients per country***

| **Country†** | **PCD patients (n)** | **Adult PCD patients (n)** | **Lobectomised PCD patients (n)** | **Adult Lobectomised PCD patients (n)** |
| --- | --- | --- | --- | --- |
| AU | 103 | 82 | 8 | 8 |
| BE | 82 | 59 | 7 | 6 |
| CH | 117 | 69 | 2 | 2 |
| CY | 36 | 30 | 6 | 6 |
| DE | 545 | 220 | 22 | 15 |
| DK | 108 | 76 | 4 | 4 |
| FR | 337 | 159 | 20 | 19 |
| IL | 141 | 78 | 4 | 4 |
| IT | 331 | 193 | 22 | 20 |
| NO | 27 | 4 | 0 | 0 |
| NL | 82 | 36 | 8 | 8 |
| PL | 171 | 55 | 27 | 19 |
| RS | 10 | 2 | 1 | 1 |
| TR | 258 | 72 | 26 | 7 |
| UK | 343 | 217 | 6 | 6 |
|  |  |  |  |  |

*As of May 2017 †AU: Australia; BE: Belgium; CH: Switzerland; CY: Cyprus; DE: Germany; DK: Denmark; FR: France; IL: Israel; IT: Italy; NO: Norway; NL: the Netherlands; PL: Poland; RS: Serbia; TR: Turkey; UK: United Kingdom

**Table S2:** Diagnostic results* for controls and lobectomised patients

|  | **Lobectomised PCD**  **(n= 130)** | **PCD Controls**  **(n=197)** | **p-value** |
| --- | --- | --- | --- |
| **Nasal Nitric Oxide (nl/min) **** | 16 (5, 40)  (n=93) | 9 (3, 17)  (n=141) | 0.003‡ |
| **TEM result** |  |  | 0.432**†** |
| No Abnormal Findings | 13/91 (14.3%) | 15/109 (13.8%) |  |
| ODA+IDA | 36/91 (39.5%) | 41/109 (37.6%) |  |
| ODA only | 20/91 (22.0%) | 31/109 (28.4%) |  |
| IDA only | 4/91 (4.4%) | 4/109 (3.7%) |  |
| IDA + MD | 4/91 (4.4%) | 10/109 (9.2%) |  |
| TUTR (8+1) | 3/91 (3.3%) | 1/109 (0.9%) |  |
| CP | 9/91 (9.9%) | 4/109 (3.7%) |  |
| Other | 2/91 (2.2%) | 3/109 (2.7%) |  |
| **HSVM result** |  |  | 0.044**†** |
| Normal | 6/107 (5.6%) | 3/152 (2.0%) |  |
| Immotile/Almost Immotile | 61/107 (57.0%) | 82/152 (53.9%) |  |
| Extremely stiff due to reduced ciliary bending | 16/107 (15.0%) | 34/152 (22.4%) |  |
| Stiff Beating pattern | 14/107 (13.1%) | 8/152 (5.2%) |  |
| Circular Pattern | 3/107 (2.8%) | 10/152 (6.6%) |  |
| Other | 7/107 (6.5%) | 15/152 (9.9%) |  |
|  |  |  |  |

*A total of 40 patients had available data on a single diagnostic test (10 lobectomised, 30 controls), 164 patients on two diagnostic tests (60 lobectomised, 104 controls) and 123 patients on three or more diagnostic tests (60 lobectomised, 63 controls). Denominators indicate number of subjects with available data for the specific parameter **Median and Interquartile Range †Pearson Chi Square Test ‡Wilcoxon Sum Rank Test TEM: Transmission Electron Microscopy, HSVM: High Speed Video Microscopy, ODA+IDA: Combined Outer Dynein Arm defect and Inner Dynein Arm defect, ODA: Isolated Outer Dynein Arm defect, IDA: Isolated Inner Dynein Arm defect, IDA + MD: Inner Dynein Arm and Microtubular Disorganisation defect, ΤUTR (8+1): Tubular Transposition defect (8+1), CP: Central Pair defect

**Table S3:** Performance of lung resection prior and after presentation at a PCD center per decade

| **Decade** | **Lung resection prior presentation at PCD center** | **Lung resection after presentation at PCD center** |
| --- | --- | --- |
| <1969 | 3 (100%) | 0 (0%) |
| 1970-1979 | 8 (73%) | 3 (27%) |
| 1980-1989 | 8 (89%) | 1 (11%) |
| 1990-1999 | 11 (48%) | 12 (52%) |
| 2000-2010 | 15 (50%) | 15 (50%) |
| >2010 | 7 (18%) | 31 (82%) |

**Table S4:** Features of 18 lobectomised patients with pre and post lung resection data. The second column presents the corresponding information for the total of 130 lobectomised PCD patients.

| **Variable** | **Lobectomised PCD**  **with pre and post lung resection data**  **(n=18)**  **Median (IQR)** | **Lobectomised PCD**  **(n=130)**  **Median (IQR)** |
| --- | --- | --- |
| Age at Presentation (n=17) | 9.8 (8.1, 11.66) | 11.5 (6.5, 20.5) |
| Current Age (n=18) | 30.7 (23.9, 32.8) | 24.9 (18.1, 40.1) |
| % Female | 8/18 (44.4%) | 72/130 (55.4%) |
| Situs Inversus (n=11) | 3/11 (27.3%) | 27/108 (25.0%) |
| Age at lung resection (n=18) | 11.8 (8.3, 13.7) | 11.9 (7.7, 16.0) |
| Lung resection prior to presentation (n=17) | 7/17 (41.2%) | 52/114 (45.6%) |
| Lung resection in childhood (n=18) | 15/18 (83.3%) | 101/121 (83.5%) |
| Frequency of lung resection performance per decade (n=18) | <1969 0  1970-1979 0  1980-1989 0  1990-1999 9  2000-2009 6  2010-2017 3 | <1969 4  1970-1979 11  1980-1989 9  1990-1999 24  2000-2009 34  2010-2017 39 |
| Extent of lung resection (n=16) | One Lobe: 14/16 (87.5%)  Two lobes: 2/16 (12.5%)  Three Lobes: 0/16 (0.0 %)  Four Lobes: 0/16 (0.0%) | One Lobe: 74/95 (77.9%)  Two lobes: 17/95 (17.9%)  Three Lobes: 2/95 (2.1 %)  Four Lobes: 2/95 (2.1%) |
| Site of lung resection** (n=17) | RML: 10/17 (58.8%)  LLL: 4/17 (23.5%)  RLL: 0/17 (0.0%)  Lingula: 3/17 (17.6%)  RUL: 0/17 (0.0%)  LUL: 0/17 (0.0%) | RML: 59/121 (48.8%)  LLL: 33/121 (27.3%)  RLL: 13/121 (10.7%)  Lingula: 9/121 (7.4%)  RUL: 5/121 (4.1%)  LUL: 2/121 (1.7%) |

Denominators indicate number of subjects with available data on the specific parameter * Full iPCD cohort that participated in the study, excluding lobectomised patients (May 2017) **Denominator reflects the total number of resected lobes. Some patients had more than one lobe resected. For one patient (with one lobe resected) the exact site was not reported.

RUL: Right Upper Lobe, RML: Right Middle Lobe, RLL: Right Lower Lobe, LUL: Left Upper Lobe, LLL: Left Lower Lobe

**Table S5:** Change in lung function and BMI over time in lobectomised PCD patients with pre and post lung resection data (n=18) and propensity score matched controls (n= 18).

| **Outcome** | **Group** | **Intercept**  **(95%CI)** | **p-value** | **Change per year**  **(95%CI)** | **p-value*** |
| --- | --- | --- | --- | --- | --- |
| FVC Z score | Lobectomised | -0.60  (-2.14, 1.39) | 0.813 | -0.080  (-0.186, 0.026) | 0.006 |
|  | Controls | -0.74  (-1.49, 0.00) |  | 0.010  (-0.03, 0.05) |  |
| FEV1 Z score | Lobectomised | -1.14  (-3.14, 0.862) | 0.753 | -0.099  (-0.222, 0.024) | 0.071 |
|  | Controls | -0.94  (-1.68, -0.20) |  | -0.029  (-0.076, -0.179) |  |
| BMI Z score | Lobectomised | -0.639  (-1.99, 0.71) | 0.116 | 0.037  (-0.036, 0.087) | 0.113 |
|  | Controls | 0.07  (-0.40, 0.54) |  | 0.006  (-0.028, 0.017) |  |
|  |  |  |  |  |  |

* P value for interaction, testing whether the relationship between independent variables (FVC, FEV_1_, BMI) and time is different between Lobectomised and Controls.
