# Supplementary figures and images for: Prevalence and course of disease after lung resection in primary ciliary dyskinesia: a cohort & nested case-control study
Source: Respir Res. 2019 Sep 18;20:212. doi: 10.1186/s12931-019-1183-y (PMC6751891; doi:10.1186/s12931-019-1183-y)

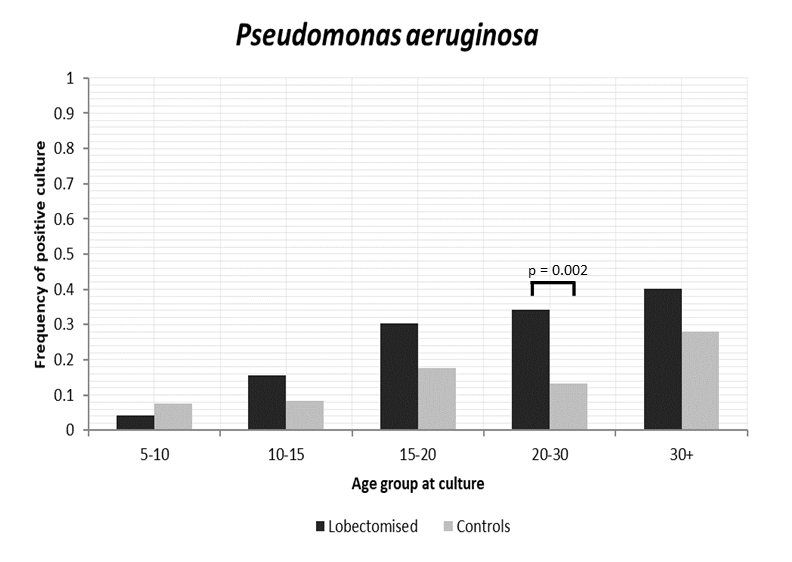

Supplement: Supplementary file 2 — Additional file 2: Figure S1. Frequency of Pseudomonas positive cultures in PCD lobectomised patients (n = 24) and controls (n = 43). Frequency of Pseudomonas positive cultures among lobectomised patients (displayed in black) and among controls (displayed in grey). The proportion of positive sputum cultures for Pseudomonas aeruginosa in each group was calculated as the sum of individual patients’ proportions of positive cultures weighted by the ratio of the number of cultures taken from the individual patient versus the total number of cultures in the specific age group. [file 12931_2019_1183_MOESM2_ESM.tif]
